# Internet of things dataset for home renewable energy management

**DOI:** 10.1016/j.dib.2024.110166

**Published:** 2024-02-10

**Authors:** Rabie A. Ramadan

**Affiliations:** a Department of Information Systems, College of Economics, Management & Information Systems, Nizwa University, Nizwa, Sultanate of Oman; b Computer Engineering Department, Faculty of Engineering, Cairo University, Giza, Egypt

**Keywords:** Smart home, IoT, Management, Renewable energy, AI, energy production, Dataset

## Abstract

Smart cities, as well as smart homes research, are becoming of concern, especially in the field of energy consumption and production. However, there is a lack in the dataset that can be used to simulate smart city energy consumption and prediction or even smart homes. Therefore, this paper provides a carefully generated dataset for smart home energy management simulation. Five datasets are generated and analysed to ensure suitability, including 20, 50, 100, and 200 homes across 365 days. For more accurate data, energy consumption and production for 50 homes are generated based on real input taken from a dataset for homes in Saudi Arabia. Due to the unavailability of a comprehensive dataset related to the complex scenario of smart home sensors, energy consumption, and peer-to-peer data exchange, synthetic data was generated to support the simulation of smart home energy generation and consumption. This synthetic data plays a crucial role in situations where simulating uncommon events, ensuring data availability, facilitating extensive experimentation and model validation, and enabling scalability are paramount. It offers a valuable opportunity to incorporate these rare yet significant occurrences into the simulation, particularly in the context of infrequent events, such as abnormal energy consumption patterns observed in smart homes. The generated data is analysed and validated in this article, ready to be used for many smart home and city research.

Specifications Table

**Table:** 

Subject	Science Data
Specific subject area	IoT data for energy management in smart cities.
Data format	Raw
Type of data	.csv file (dataset with numbers)
Data collection	This is synthetic data generated to reduce the gap in the unavailability of IoT energy management in smart homes. Virtual datasets with made-up but statistically relevant properties are created to mimic actual data. Due to many variables, understanding the effects of individual elements in complex energy systems like smart homes or solar energy production is challenging. Part of the dataset uses one of the weather data as a base for home energy generation and consumption.
Data source location	The data is based on northern Saudi Arabia Hail, Saudi Arabia.
Data accessibility	Repository name: Mendeley Data identification number: 10.17632/5h86vnxw68.1 Direct URL to data: https://data.mendeley.com/datasets/5h86vnxw68/1 Instructions for accessing these data: the data is available as .csv files that can be directly downloaded [1].

## 1 Value of the Data

• Why are this data valuable?
• The dataset is valuable due to the unavailability of home energy data captured of IoT device with varied number of homes.
• How can these data be reused by other researchers?
• The data can be used to simulate the energy management and prediction in smart cities. The details and the analysis of the data are provided in subsequent sections.

## 2 Background

The lack of data is a typical real-world problem caused by privacy issues, complex circumstances, logistical restrictions, or costly data collecting and storage costs. Emerging areas of inquiry or fresh implementations with insufficient data often face this difficulty. In fact, there is a huge amount of research on smart cities and smart homes. However, implementing a real smart city or even a smart home is a complex problem that needs effort and is costly. Therefore, most researchers use simulators to tackle many of the problems. One of the critical problems is predicting home energy consumption and production and buying and selling energy from others.

Another problem is the security of peer-to-peer energy transfer between homes in smart cities, for example. Therefore, there is a need for a large dataset that could be used to emulate smart homes' energy consumption and prediction. Unfortunately, we could not find such data, especially for a large number of homes. That is why the dataset described here is synthetically generated and verified for the research community to tackle many of these field problems. The only difference between the synthetically generated data and the real data is that the synthetical data is generated based on real data, just to increase the data volume and to be able to simulate the smart cities energy consumption and production.

Some of the work has been done in this area, but the dataset used is either very small or not comprehensive enough to include all the required fields. For instance, in [2], the authors covered HEMS control techniques, optimization algorithms, and advanced metering infrastructure (AMI) that allow this integration. In [3], ZigBee-based energy devices are used to monitor household appliances and light energy usage. Similarly, in [4], it creates smart energy-based home device descriptions and standard practices for demand response and load control "Smart Energy" applications. On the other hand, the authors in [5] investigated and used deep learning-based strategies for energy consumption in smart residential buildings. A two-stage home energy management system that considers the best energy use is proposed in [6], as well as the reliability of power patterns and how the building's temperature changes over time. The used datasets in those papers are made specific for the proposed methods or just a few samples for prototype examination. Therefore, as can be seen, many applications can benefit from the proposed dataset. For instance, it can be used for analysing smart home energy consumption [1] or smart home energy management and prediction [3], [4], [5], [6], [7]. It also could be utilized to answer how smart home technologies can contribute to more sustainable energy use. It may explore consumer perceptions and the effectiveness of smart home devices in reducing energy consumption [8]. Moreover, the dataset could be used for predictive analysis for residential electricity consumption [9], where it might utilize smart meter data to predict residential electricity consumption patterns, providing insights into how energy use varies seasonally or in response to different factors. Furthermore, one may use the dataset for machine learning to predict and schedule energy consumption [10].

Synthetic data may bridge data accessibility gaps. Virtual datasets with made-up but statistically relevant properties are created to mimic actual data. Synthetic data is essential for experimentation and model validation. Due to many variables, understanding the effects of individual elements in complex energy systems like smart homes or solar energy production is difficult. Synthetic data lets researchers modify and isolate variables for precision testing and model refining. Flexible synthetic data allows systematic model validation across various situations, improving model robustness and generalizability.

The generated dataset will have a great impact on research in the field of smart cities, where smart homes use solar cells for energy generation. At the same time, they may buy from others if their generated energy is insufficient. Peer-to-peer energy exchange research can also benefit from the generated dataset. Moreover, the dataset will help in energy prediction in northern areas of Saudi Arabia as well as in similar regions.

Although the generated dataset could help research study energy forecasting, management, and peer-to-peer energy exchange in smart cities, smart cities could have more than 200 homes. The current dataset serves small, medium, and large-scale smart cities. However, the large-scale smart cities are limited to 200 homes.

## 3 Data Description

This section discusses five datasets, including 20, 50, 100, and 200 homes across 365 days, that are generated and made available at the data repository [1]. The reason behind dividing the dataset into four categories is the interrelation between the homes in each category. In other words, the production data in each file is generated within a certain percentage of the consumption data to minimize the variations and outliers in the data. At the same time, the data is categorized to study different types of energy prediction and management in small, medium, and large-scale smart cities. However, the files can be merged in case there is a need for a large number of homes, but careful attention must be paid to the merge sequence. Also, it has been assumed that homes in smart cities consume energy and produce energy simultaneously; therefore, they can exchange and trade energy with each other. The energy produced from different sources is summed and saved in the production files in the dataset, in which the main concern of the dataset is the energy produced per home, regardless of its source.

The energy consumption files for 20, 50, 100, and 200 homes are named 20_consumption_data.csv, 50_consumption_data.csv, 100_consumption_data.csv, 200_consumption_data.csv. Also, there is a consumption data file generated based on sessional data from northern Saudi Arabia for 50 homes, named 50_consumption_Sessional_data.csv. Sessional data refers to the summer session in Saudi Arabia, where the weather fluctuates. For dataset generation, actual meteorological data from Saudi Arabia is used to build the fifth dataset for 50 residences. These datasets track energy usage from typical household appliances. In particular, energy usage data covers fridges, lights, ACs, and kitchen equipment. Each row in the dataset corresponds to a specific day, while each column represents the energy consumption of a particular home on that day. Positive values indicate energy consumption from the grid or other sources, with the magnitude representing the amount of energy consumed. The dataset reflects the variability in energy consumption patterns among the homes, with some consistently consuming energy throughout the 365 days while others exhibit fluctuations between energy consumption and surplus energy production.

Like the consumption files, the corresponding production files are generated, namely, 20_production_data.csv, 50_ production_data.csv, 100_production_data.csv, and 200_ production_data.csv.

**Fig. 1:**
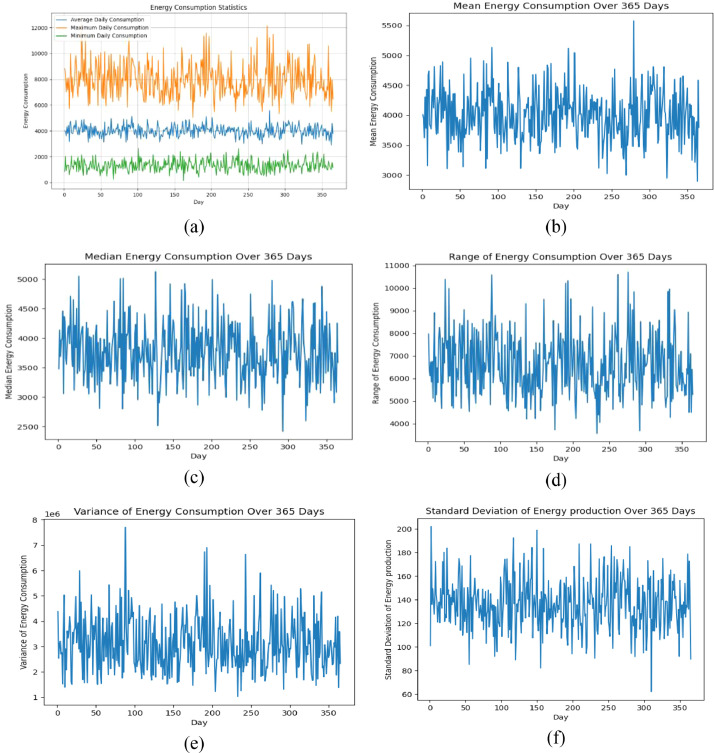
Energy consumption statistics for 20 homes, (a) average, minimum, maximum daily consumption, (b) mean, (c) median, (d) range, (e) variance, (f) standard deviation.

The Mean, Range, Median, Variance, and Standard Deviation are conducted on the 20 homes dataset to verify the validity of the generated dataset. Fig. 1(a) shows the average, maximum, and minimum daily consumption in terms of Watts. As seen in the figure, there are variations in the daily consumption between homes, which makes the data suitable for testing the proposed algorithms' efficiency. The provided data gives insights into energy consumption over a given period. The mean energy consumption, Fig. 1(b), ranges from 2894.5 kWh to 5576.1 kWh, representing the average energy usage during the observed days. The median energy consumption, shown in Fig. 1(c), ranges from 2419.79 to 5126.63. The median represents the middle value in a sorted dataset and provides a measure of central tendency. Similar to the mean, the median values indicate values below and above the central point, respectively. The Range of energy consumption, presented in Fig. 1(d), varies from 3563.47 to 10707.62, indicating the difference between the highest and lowest values. A wider range suggests a greater variability in energy consumption levels during the observed period. The variance of energy consumption, presented in Fig. 1(e), ranges from 1024347.62 to 7703270.19, representing the variability or dispersion of the data points from the mean. A higher variance indicates a greater spread of energy consumption values around the average. The standard deviation, depicted in Fig. 1(f), ranges from 1012.1 to 2775.48, providing a measure of the dispersion or variability in energy consumption. A higher standard deviation implies a wider spread of values around the mean, indicating greater volatility or fluctuation in energy usage.

## 4 Experimental Design, Materials and Methods

### 4.1 Experimental Design

The main goal of this dataset is to provide highly accurate synthetic data for energy consumption and production for real-world scenarios in smart cities. To achieve this objective, we designed a simulator with the following key components:

• Input Parameters: The simulator begins with a careful selection of the input parameters. These parameters involve the number of homes, appliance parameters (including fridges, AC units, lighting, and kitchen appliances), energy generation parameters (solar and wind), and the Range of energy values for potential consumption and production. These values are based on robust data derived from real weather datasets collected from the northern region of Saudi Arabia. This ensures that the simulator mirrors actual environmental conditions.
• Programming Language: The simulator is designed using Python programming language. Python is well known for its extensive data handling, manipulation, scientific computing, and statistics libraries. Its capabilities perfectly fit the requirements of data generation.

### 4.2 Materials

The materials used in the dataset generation are mainly computational resources, including high-performance computers with Python and the relevant installed libraries. Also, a real weather dataset captured from the northern region of Saudi Arabia, Hail City, was used to drive the input parameters of the simulator to satisfy the real-world condition data generation.

### 4.3 Methods

• Initialization of Parameters: The generation of the dataset commences with the initialization of input parameters, which encompass the number of homes, appliance parameters, and energy generation parameters. Those parameters serve as the base for generating the synthetic data that faithfully produces actual energy consumption and production of homes in smart cities. In each home, the fridge is assumed to run 24/7, and the lights are on for 6 hours. The AC runs for 5 hours daily, and the kitchen appliances for 3 hours only.
• Appliance Energy Consumption Modeling: The simulator proceeds to model home appliances such as fridges, air conditioning units, lighting, and kitchen appliances. The energy consumption of these appliances is generated randomly based on carefully selected variables. The mean values and standard deviation are estimated based on the historical weather data collected from the northern region of Saudi Arabia. This technique ensures that variations in usage patterns are realistically and truly influenced by weather conditions based in the northern region of Saudi Arabia.
• Energy Balance Calculation: The daily energy for each home is calculated by subtracting energy generated from solar and wind sources from the home-consumed energy.
• Simulation of Solar and Wind Energy Generation: In parallel, the simulator accurately generated the solar and wind energy using the provided data and the initial values of the input parameters. Solar production is simulated using a normal distribution with a mean of 1000 and a standard deviation of 100. Wind production values are normalized with a mean of 900 and a standard deviation of 100, accounting for variations in wind patterns. This approach perfectly captures daily fluctuations in renewable energy production according to our statistics analysis of the generated data.

## Limitations

However, this dataset has some limitations; for instance, still 200 homes are at the border of a large-scale number of homes in smart cities and cannot reflect the whole concept of smart cities. Also, although the data is based on realistic consumption for home appliances, the details of the energy consumption of each appliance are not provided in the data; only the overall energy consumption of the home per day is provided. Moreover, the produced energy is given as a value regardless of how many solar devices are available for each home. However, these limitations could be handled in the updated dataset version.

## Ethics Statement

The authors have read and follow the ethical requirements for publication in Data in Brief and confirming that the current work does not involve human subjects, animal experiments, or any data collected from social media platforms.

## CRediT authorship contribution statement

**Rabie A. Ramadan:** Conceptualization, Methodology, Software, Data curation, Writing – original draft, Visualization, Investigation, Software, Validation, Writing – review & editing.

## Data Availability

Energy Consumption Dataset For Smart Homes (Original data) (Mendeley Data).
